# Turning Analysis during Standardized Test Using On-Shoe Wearable Sensors in Parkinson’s Disease

**DOI:** 10.3390/s19143103

**Published:** 2019-07-13

**Authors:** Nooshin Haji Ghassemi, Julius Hannink, Nils Roth, Heiko Gaßner, Franz Marxreiter, Jochen Klucken, Björn M. Eskofier

**Affiliations:** 1 Machine Learning and Data Analytics Lab, Department of Computer Science, Friedrich-Alexander-University Erlangen-Nürnberg (FAU), Carl-Thiersch-Strasse 2b, D-91052 Erlangen, Germany; 2 Department of Molecular Neurology, University Hospital Erlangen, Schwabachanlage 6, D-91054 Erlangen, Germany

**Keywords:** Parkinson’s disease, pathological gait, turning analysis, wearable sensors, mobile gait analysis

## Abstract

Mobile gait analysis systems using wearable sensors have the potential to analyze and monitor pathological gait in a finer scale than ever before. A closer look at gait in Parkinson’s disease (PD) reveals that turning has its own characteristics and requires its own analysis. The goal of this paper is to present a system with on-shoe wearable sensors in order to analyze the abnormalities of turning in a standardized gait test for PD. We investigated turning abnormalities in a large cohort of 108 PD patients and 42 age-matched controls. We quantified turning through several spatio-temporal parameters. Analysis of turn-derived parameters revealed differences of turn-related gait impairment in relation to different disease stages and motor impairment. Our findings confirm and extend the results from previous studies and show the applicability of our system in turning analysis. Our system can provide insight into the turning in PD and be used as a complement for physicians’ gait assessment and to monitor patients in their daily environment.

## 1. Introduction

Gait is an important part of mobility that is impaired in neurodegenerative diseases like Parkinson’s disease (PD). As the disease progresses, gait fluctuations become more severe. Different locomotor patterns in gait, such as straight walking and turning, require different levels of functioning and coordination. For a person with impaired mobility caused, for example, by PD, turning is challenging and potentially risky, even more than straight walking [1,2]. There have been attempts to identify and characterize turning abnormalities in order to complement the physicians’ assessment of pathological gait.

Studies showed that turning deficits are manifested in mild PD even when there are no signs of impairment in straight walking [3]. Difficulty while turning may lead to posture instability and, potentially, even falls [4,5]. Risk of falling is higher during turning compared with straight walking [4,5]. Furthermore, deterioration of motor function during turning can cause progressive episodes of freezing of gait (FoG) [6,7,8].

Some studies have attempted to utilize the definition of disease stages and motor impairments by UPDRS-III [9] and H&Y [10] clinical scores and objectively assess turning deficits [11,12,13,14,15]. Studies on spatio-temporal parameters quantifying turning have demonstrated decreased speed, longer duration of turning, and a larger number of strides as the disease progresses [3,16,17,18]. Postural stability also decreases during turning for PD patients in comparison to healthy controls, particularly during fast walking [19].

Outside the clinics and in the majority of standardized clinical tests, a gait sequence includes both straight walking and turning. In order to differentiate between them during the course of a gait, different definitions of turning have been presented in the literature. For example, turning was defined as the movement between two pre-defined points that indicated the initiation and termination of turning [5]. Salarian et al. [17] used mathematical modeling in order to isolate turns from the whole gait sequence. Spatio-temporal parameters extracted from individual strides are different in straight walking compared to turning. Many studies used characteristics and statistics of spatio-temporal gait parameters to define turning [3,6,20]. Without a standard turning definition, studies then presented some clinical validations to support their definitions—for example, they showed that turning parameters were correlated to the established clinical scores [3,6,20].

Gait and turning can be measured by a variety of systems—from accurate but stationary motion capture systems [19] to small wearable sensors [3,17]. The focus of this study is on wearable sensors, since they give the opportunity to perform long-term monitoring of PD patients. Sensor placement plays a crucial factor in designing wearable systems. Many turning studies place the sensors on the upper extremity [3,6,20]. One advantage is that turning is easily detectable in the sensor signals [17]. However, gait disturbances such as FoG cannot be detected clearly from sensors on the upper extremity. Such systems still need additional sensors on the lower extremity in order to quantify turning in terms of spatio-temporal parameters [3,17]. In contrast, sensors on the lower extremity and, in particular, on the shoe provide higher biomechanical resolutions. Panebianco et al. [21] examined different sensor locations and showed that as sensors get closer to the foot, higher accuracy for gait events and parameters can be obtained. Moreover, for long-term monitoring of patients, sensors integrated in the footwear are less obtrusive and stigmatizing.

In order to measure gait, we used wearable sensors mounted on the lateral side of the shoe. In order to isolate turning from gait, we used the statistics of spatio-temporal parameters. The goal of this study is to show the applicability of the system in the objective analysis of turning and to evaluate whether it confirms the findings of other studies. To this end, we first introduce our novel turning isolation algorithm targeting data from a standardized 4×10 m gait test measured with wearable sensors placed on the shoe. Then, we quantify the isolated turnings through several spatio-temporal parameters that proved to be effective in detecting pathological gait [22,23,24]. Through meticulous statistical analysis, we evaluate the turning abnormalities in a large PD cohort. The value of this objective turning assessment was clinically validated by the correlation of the turn-derived parameters to clinical scores, including motor impairment and disease stages in PD.

## 2. Methods

### 2.1. Wearable Measurement System

**Figure 1:**
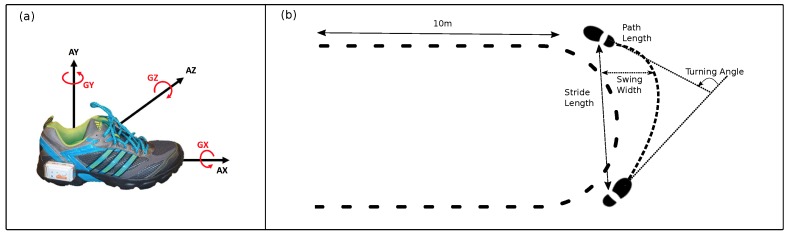
(**a**) Shimmer sensor placement and axes definition. (**b**) Definition of turning angle, stride length, path length, and swing width.

For our experiments, data was recorded with a Shimmer 2R/3 Inertial measurement unit (IMU) (Shimmer Sensing, Dublin, Ireland), measuring acceleration and angular velocity at 102.4 Hz. Each unit consisted of a tri-axial accelerometer (range Shimmer 2R: ±6 g, Shimmer 3: ±8 g) and a tri-axial gyroscope (range Shimmer 2R: ±500${}^{\circ }$/s, Shimmer 3: ± 1000${}^{\circ }$/s). The sensor units were mounted laterally on each shoe below the patient’s ankle. The measurements from both feet were included in the experiments. Figure 1 shows the sensor placement on the shoe and the axes definition.

### 2.2. Study Population

We recruited 108 PD patients during their regular visit in the movement disorder outpatient center at the University Hospital Erlangen. Sporadic PD was defined according to the guidelines of the German Association for Neurology (DGN), which are similar to the UK PD Society Brain Bank criteria [25]. Patients had to be able to walk independently (H&Y <4, UPDRS gait item <3) [10,26]. All PD patients were clinically (UPDRS-III) and biomechanically (gait analysis) investigated in stable ON medication without the presence of clinically relevant motor fluctuations during the assessments. We had an exclusion criterion for a severe cognitive impairment. To obtain quantitative gait data from controls, we recruited 42 age-matched controls with no signs of PD and/or other motor impairments. With respect to age, height, and body-mass-index (BMI), PD and control cohorts were matched (see Table 1). Data regarding laterality of the disease can be found in Table 1, where the UPDRS sub-items of rigidity lower and upper extremities were reported. This data shows that patients affected on the right and left sides are almost equally represented in our cohort. Written informed consent was obtained from all participants (IRB-approval-No. 4208, 21.04.2010, IRB, Medical Faculty, Friedrich-Alexander University Erlangen-Nürnberg, Germany).

Participants walked freely at a comfortable, self-chosen speed in an obstacle-free and flat environment for 4×10 m. After each 10 m of straight walking, participants were instructed to turn $180^{\circ }$ at a preferred direction.

### 2.3. Turning Isolation

The standardized 4×10 m walking included four straight gait bouts and three turnings in between each two straight bouts. The goal was to isolate the three turnings from the whole gait sequence. To this end, the gait sequence was segmented to individual strides semi-automatically [27,28]. These strides should then be categorized as straight walking, turning, and transitions between straight walking and turning. In order to differentiate between these categories, we used statistics of spatio-temporal parameters.

The change of azimuth between two successive mid-stances was defined as the turning angle between consecutive strides (see Figure 1). The absolute values of turning angles were considered since the sign of values only showed the direction of the turnings, which is not of importance in our analysis. Similarly to Mariani et al. [20], strides with turning angles larger than $20^{\circ }$ were classified as turning.

In order to identify transition strides in a gait sequence [20], again, statistics over turning angles were used, since this parameter is the best indicator of spatial foot movement during turning (see Figure 1). The turning strides with angles larger than $20^{\circ }$ were eliminated from the sequence. A gamma distribution was then fitted to the tuning angels from the rest of the strides. We chose gamma distribution due to the fact that the distribution is one-hand tailed, in a way that strides from straight walking mainly centers on the mean. The highest 10% of the distribution was classified as the transition if the strides were adjacent to the turning strides. In fact, the strides in the highest 10% of the distribution were considered as anomalies in the distribution of straight strides. For turning analysis, we only considered turning and transition strides.

### 2.4. Turning Parameters

After the turning isolation, we had three sets of strides related to three turns in the standardized test. We extracted spatio-temporal parameters from these strides based on the algorithms in previous works [20,22]. The algorithms for obtaining parameters from our wearable sensor-based system were validated previously using a gold standard, such as an optical motion capture system or instrumented walkway. To quantify turning, two sets of parameters were computed for each turning—per-stride parameters and global parameters per-turn.

For the first group, a set of parameters was extracted from each stride: stride time, path length (normalized on patient’s height), stride length (normalized on patient’s height), stride velocity, and swing width. In turning, it is very likely that a stride has a curved trajectory, rather than a straight line. In such cases, length of movement in the straight line between the beginning and end of a stride is measured as stride length. In addition, path length was introduced to measure curve length between the beginning and end of a stride (see Figure 1). All these parameters were calculated from mid-stance of a stride to the successive mid-stance.

For the global parameters, we calculated the number of strides and total duration per turn. This set of parameters measures characteristics of the whole turn.

### 2.5. Statistical Analysis

In order to determine whether parameters can distinguish between different groups (controls and three stages of disease (see Table 1)), we applied the one-way analysis of variance (ANOVA). When a significant difference was found, a post hoc analysis was performed using Bonferroni’s test to obtain a pairwise comparison between the groups. The significance level was set at p<0.05. For measuring effect sizes, $\eta^{2}$ was defined as the ratio of variability between groups to the total variation in the data that was used. Cutoff values for small, medium, and large effect sizes were set at 0.01, 0.06, and 0.14, respectively, according to Cohen [29]. Statistical analysis and parameter computations were performed using MATLAB R2015a.

## 3. Results

As the disease progresses, gait impairment associated with deteriorated mobility becomes more prevalent. In this section, we examined whether spatio-temporal parameters that characterize turning were able to reflect gait impairments.

**Figure 2:**
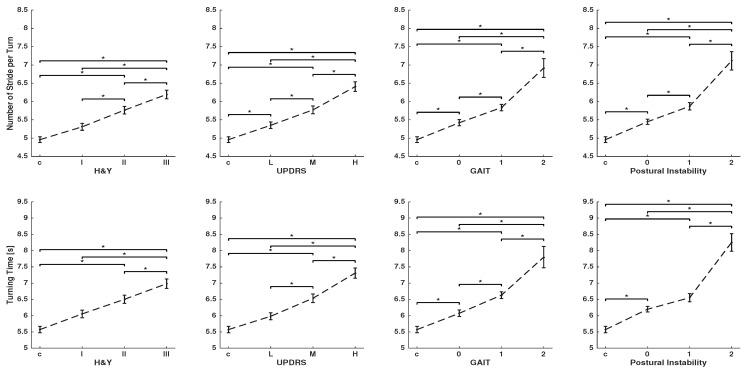
Global parameters characterizing turning: number of strides per-turn and turning time were calculated for controls and PD patients grouped according to H&Y disease stage, UPDRS-III total score, and the single items, gait and postural instability of the UPDRS-III. Group data are displayed as mean ± SEM and were compared using one-way ANOVA followed by Bonferroni’s post hoc test, where * indicates p<0.05.

**Figure 3:**
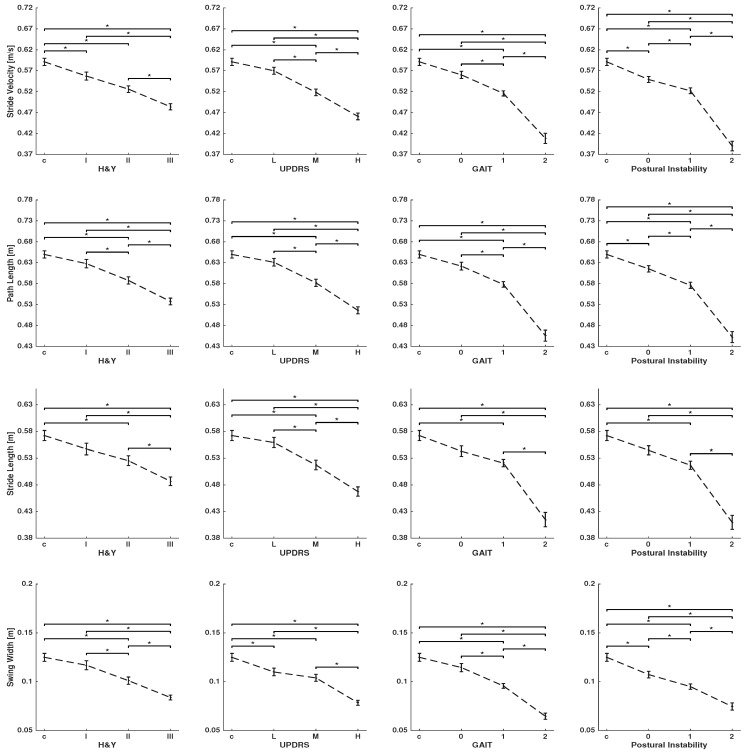
Per-stride parameters characterizing turning: stride velocity, path length, stride length, and swing width were calculated for controls and PD patients who were grouped according to the H&Y disease stage, UPDRS-III score, and the single items, gait and postural instability, of the UPDRS-III. Group data are displayed as mean ± SEM and were compared using one-way ANOVA, followed by Bonferroni’s post hoc test, where * indicates p<0.05.

Figure 2 and Figure 3 show spatio-temporal parameters that are characteristic of turning for global and per-stride parameters, respectively. Clinical scores in PD studies determine the severity of gait impairment and disease stages: the H&Y, UPDRS-III score, and the UPDRS-III sub-items for gait and postural instability. Patients with different levels of disease severity (see Table 1) and controls were statistically compared using ANOVA, followed by Bonferroni’s post hoc test.

As the disease progresses, stride velocity, path length, stride length, and swing width (per-stride parameters) decreases, and as a result, patients need more strides and time (global parameters) to complete a turn. This can be observed for all clinical scores, although the two sub-items of gait and postural instability are showing larger differences between stages of the disease. Stride time shows no clear change between different groups.

Global parameters showed that PD patients, in contrast to controls, need significantly more time and a larger number of strides to complete a turn (see Figure 2). Number of strides per turn, in particular, shows a significant difference between the control and even early stage of the disease for the UPDRS-III score and its two sub-items. Moreover, there are significant differences between stages of the disease in most comparisons. Per-stride parameters, except stride time, show a significant difference between the controls, mild, and severe stages of the disease for all clinical scores. Stride velocity, stride length, path length, and swing width are able to differentiate disease severity by means of all tested clinical scores (see Figure 3).

To quantify effect sizes, $\eta^{2}$ is reported in Table 2. The effect sizes range from small to large. The largest effect sizes are obtained consistently over all clinical scores with p<0.001 by the global parameters, number of strides per turn, and turning time. Path length showed consistently higher effect sizes than stride length, which suggests that it is a more meaningful parameter for estimation of spatial foot displacement in turning. The effect sizes of per-stride duration are very small.

## 4. Discussion

The aim of the present study was to investigate whether an on-shoe, sensor-based gait analysis system reflected turning abnormalities and whether it could objectively complement physicians’ gait assessments. To this end, we recruited 108 PD patients and 42 age-matched controls, and measured their gait during a 4×10 m walk by using our system. We then isolated the turnings from the whole gait sequence and quantified them using several spatio-temporal parameters. The parameters extracted using an on-shoe wearable system were previously validated against gold-standard systems, such as an optical motion capturing system [30] or instrumented walkway [22], and results indicated their technical validity. The clinical validation that followed turn quantification showed that turning parameters extracted using our measurement system and the turn isolation algorithm can effectively reflect gait abnormalities and be successfully used for the objective assessment of turning.

There have been many studies regarding turning analysis in PD [3,17,20]; yet, there is no unique way to define turning. Turning has been defined using mathematical modeling [17], statistics of spatio-temporal parameters [20], or the path between two pre-defined points [5]. One reason for these diverse turning definitions is that, basically, there is no standard way to determine the start and end of the turning. Common gold standards, such as motion-capture systems or videos, cannot provide a ground truth for turning. Since transitions between straight walking and turning happen gradually, it is inherently difficult to determine a specific start- and end-point for turning. A technical validation seems impractical with the usual gold standards. Nevertheless, a specific definition of turning, supported by some clinical validations that show its usability, can be an asset in objective gait assessment [3,17,20].

Turns can have different lengths, angles, and bases of support. We can expect that different types of turning require different levels of coordination [19]. In this study, we analyzed $180^{\circ }$ during the 4×10 m walk test. Turnings with $180^{\circ }$ were also analyzed in other standardized tests, like Timed Up and Go (TUG) [16,31,32]. We studied a 4×10 m walk because it includes three turns, which makes it statistically more meaningful to draw any general conclusions from the experiments. Regardless of the type of the turns, the underlying concepts that were used in this study are valid, although the turning isolation algorithm may need some adjustments to distinguish between straight walking and turning in an optimal way.

The findings of this study confirm the results from other studies [11,12,13,14,15], showing that spatio-temporal parameters can manifest gait deficits even in early stages of the disease. Results show that as the total duration of a turn increases, the stride length and velocity decreases and more strides are needed to complete a turn in the PD population. Such changes in parameters were scaled with PD severity. Global parameters of turning, such as the number of strides per turn and the total duration of the turn, can distinguish different groups. This is an important finding for PD studies, because gait problems are difficult to detect by physicians in early stages of the disease, whereas sensor signals can capture subtle differences between a healthy and abnormal gait in the early stages of the disease. The large effect sizes for global parameters further emphasized the efficiency of these parameters for yielding statistical differences between different groups. Previous studies showed similar results for such global turning parameters [3,16,17]. Per-stride parameters of stride velocity, path length, stride length, and swing width can distinguish the majority of groups, although to a lesser extent in contrast to global parameters. For example, the distinction between controls and early-stage PD patients is more effective in global parameters. Furthermore, the effect sizes for per-stride parameters are in the range of small to medium (see Table 2), which again proves to be less effective than global parameters.

The total duration of turns showed a clear correlation with clinical scores, but such a correlation has not been obtained for per-stride timing. We may be able to explain this by considering two kinds of compensatory actions taken by patients in order to complete the turning. One compensatory action is to take smaller strides, and the other one is having longer pauses in a mid-stance phase in order to secure balance. While the first compensatory action decreases the per-stride time, the latter increases it. These compensatory actions may be different from patient to patient, and a patient may take both of these actions to safely complete a turn. Hence, overall, we cannot see any clear increase or decrease in the per-stride duration; however, the total turn duration did increase, because we may have a decrease of time per stride but patients take more strides that compensate for the decrease in time per stride. Having a long pause at mid-stance phases did not have any effect on stride and path length. These parameters decrease as the disease progresses.

Established clinical scores have no sub-item to assess specific characteristics of turning. Turning is evaluated as part of the gait in general; yet, our findings show that clinical scores reveal turning deficits at different levels. Parameters consistently show a higher correlation with gait and postural instability sub-items than with H&Y and UPDRS-III global scores, both in terms of p-values and effect sizes. Postural instability and gait sub-items are widely used for assessing gait, balance, and risk of falling in PD patients [23]. These two sub-items effectively demonstrate turning abnormalities, even at early stages of the disease (see Figure 2).

Despite the importance, there has not been a study to objectively compare straight walking and turning parameters in order to understand which set of parameters reflects gait abnormalities better. However, parameters quantifying straight walking differentiate between controls and PD patients in more moderate stages of the disease or higher levels of motor impairment [23]. Spatio-temporal parameters characterizing gait abnormalities have been widely used in data-driven applications, from PD diagnosis to disease monitoring [20,33]. However, most of such studies focus only on analyzing straight walking. Our results suggest that turning analysis may improve the performance of data-driven methods in medical applications.

One of the key goals of mobile gait analysis is to monitor patients outside of the clinics. Long-term monitoring of patients during the course of a day can provide better insight into their disease condition, in contrast to time-limited examinations inside the clinics [3]. Moreover, continuous monitoring of patients can be supplemented with preventative strategies for falling and FoG. The fact that turning during standardized tests demonstrates clear signs of deficiency emphasizes that turning analysis needs to be integrated into the long-term gait analysis. Turning isolation during long-term monitoring is even more challenging than in a standardized test, since the strides can be highly variable and different types of turning may happen within the course of a day. Some studies successfully addressed turning analysis in long-term monitoring [3,6], although they did not use on-shoe sensor systems. More research is needed to understand how findings of the current study can be transferred using an on-shoe sensor system to long-term monitoring.

Laterality of PD is another important factor in turning analysis, since turning to the direction of the most affected side is more challenging for patients. However, analyzing the laterality of the disease was beyond the scope of this study—here, the patients were instructed to turn at a convenient speed and preferred direction.

A limitation of our study is that we were, at this stage, not able to analyze the asymmetry between the left and right foot, since the sensors were not synchronized. Even better results may be obtained by an experiment design that takes into account the specific characteristics of PD patients and assessments during OFF medication.

## 5. Conclusions

Mobile gait analysis using wearable sensors offers elaborate assessments of pathological gait, leading to deeper insight into the motor deficits of PD. A high level of deficiency has been frequently reported for turning in PD. We investigated the feasibility of turning analysis during standardized gait tests using on-shoe wearable sensors. Turning measurements in our experiments clearly demonstrated turning deficits in Parkinson’s patients. However, global parameters proved more effective than per-stride parameters. This should be taken into account in designing gait analysis systems, and has an important implication for PD clinical examinations, since physicians can readily assess global parameters. The current result is in alignment with other studies of turning in Parkinson’s patients, which proves the feasibility of turning analysis using on-shoe sensor systems. The results of the current study can be applied to studies evaluating turning inside the clinic, and provide useful insight into long-term monitoring outside the clinic.

## Figures and Tables

**Table 1:** Clinical characteristics of patients with Parkinson’s disease (PD) and healthy controls.

	PD (N = 108)	Control (N = 42)
Age (years)	57.61 ± 10.42 [36–85]	58.78 ± 11.14 [41–84]
Sex (Male/Female)	74/34	25/17
Height (m)	1.74 ± 0.1	1.73 ± 0.07
BMI	25.81 ± 3.71	26.48 ± 3.76
Hoehn and Yahr stage	2.06 ± 0.84	
I (<1)	28	
II (1-2]	34	
III (2<)	46	
UPDRS-III total	18.24 ± 9.8 [2–50]	
Low [0–12]	36	
[13–22]	38	
High [23<)	34	
Laterality based on Rigidity item (upper and lower extremity)		
No rigidity or both sides	22%	
Right side	42%	
Left side	36%	
Gait item		
0 [0]	34	
1 (0–1]	62	
2 (1–2]	12	
Postural stability item		
0 [0]	46	
1 (0–1]	49	
2 (1–2]	13	

**Table 2:** ANOVA test: $\eta^{2}$ values for different parameters and clinical scores. Values with * correspond to p<0.001. Bold font indicates values with strong effect sizes.

Parameters	H&Y	UPDRS	Gait	Postural Instability
Number of Strides per-Turn	**0.172** *	**0.2** *	**0.202** *	**0.232** *
Turning Time	**0.149** *	**0.199** *	**0.187** *	**0.228** *
Stride Velocity	0.054 *	0.057 *	0.06 *	0.069 *
Path Length	0.054 *	0.054 *	0.06 *	0.063 *
Stride Length	0.03 *	0.03 *	0.034 *	0.038 *
Mid Swing	0.034 *	0.035 *	0.039 *	0.029 *
Stride Time	0.003	0.003	0.002	0.007 *
